# A young Asian woman with thrombosis in the right renal vein and inferior vena cava: A rare case report and literature review

**DOI:** 10.1097/MD.0000000000032121

**Published:** 2022-12-02

**Authors:** Jhe-Yuan Hsu, Tzong-Shiun Li

**Affiliations:** a Division of General Practice, Department of Medical Education, Changhua Christian Hospital, Changhua, Taiwan; b Department of Cardiovascular Surgery, Changhua Christian Hospital, Changhua, Taiwan.

**Keywords:** case report, COVID-19 vaccine, infertility, renal veins thrombosis, ultrasound

## Abstract

Renal vein thrombosis (RVT) is a rare clinical entity wherein a blood clot forms in one of the major renal veins or its tributaries. Patients with certain risk factors, such as adults with nephrotic syndrome and infants with dehydration, are susceptible to developing it.

**Patient Concerns:**

We present a young woman with primary thrombosis in the right renal vein and inferior vena cava. Hormonal therapy and the reception of the Medigen Vaccine Biologics Corporation coronavirus disease 2019 vaccine were the suspected risk factors for developing this disease.

**Primary Diagnosis, Interventions, Outcomes:**

The primary thrombosis in the right renal vein and inferior vena cava was diagnosed using abdominal computed tomography (CT), and 90% of the thrombus in the right renal vein was dissolved after ultrasound-assisted catheter-directed thrombolysis followed by urokinase infusion for 1 week. Antibiotics and rivaroxaban were prescribed for 3 days and 5 months, respectively. Cryoprecipitate transfusions based on the level of fibrinogen were also prescribed. No long-term complications were noted in the clinic visits. We demonstrate the results of ultrasound-assisted catheter-directed thrombolysis using urokinase infusion for thrombosis in the right renal vein and inferior vena cava. Lastly, we review the literature discussing RVT relevant to this case.

**Conclusion:**

This study reveals the successful use of the novel technique, ultrasound-assisted catheter-directed thrombolysis using urokinase infusion, for the treatment of RVT.

## 1. Introduction

Renal vein thrombosis (RVT) is caused by the formation of a thrombus in the renal veins or their tributaries.^[1]^ The reported rate of RVT varies widely, likely owing to the selection of patients and detection methods used in studies. In one study, patients without membranous nephropathy who underwent venography showed a prevalence of RVT ranging from 10% to 50%, depending on the underlying disease.^[2]^ In another study, patients with membranous nephropathy showed a higher RVT prevalence, from 20% to 60%.^[3]^ Spontaneous RVT is quite rare in ambulatory individuals without nephrotic disease or underlying renal malignancy.^[4,5]^ According to previous literature, the precipitating factors for RVT include trauma, oral contraceptive use, hypovolemia, inherited thrombophilia, and extrarenal compression of the renal vein; another suspected factor is coronavirus disease 2019 (COVID-19).^[6,7]^ In this article, we present the case of a young woman with primary thrombosis in the right renal vein and inferior vena cava following hormone therapy for infertility and vaccination for COVID-19. Additionally, we propose a therapy protocol and review the literature for RVT.

## 2. Case presentation

**Figure 1.**
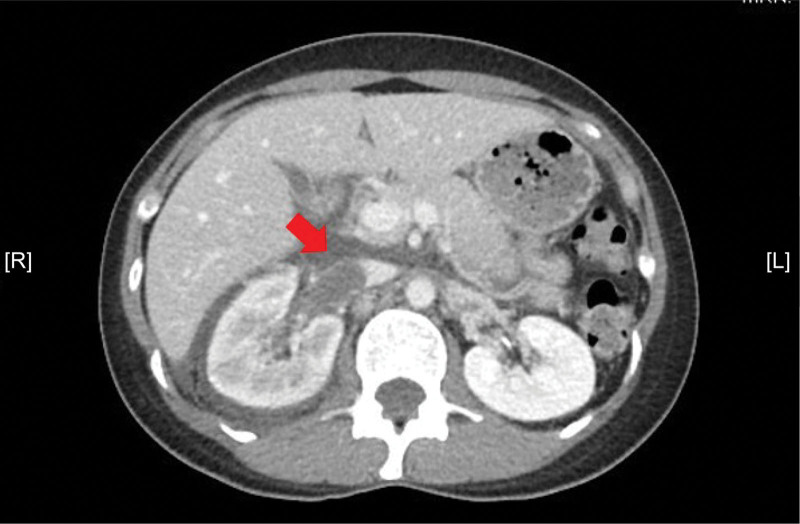
Contrasted abdominal CT images showing thrombi within the right renal veins and inferior vena cava. CT = computed tomography.

A 41-year-old Taiwanese woman with a history of infertility, depression, and insomnia, controlled by medications, presented with right flank pain for 2 days. The patient was fully ambulatory when she was admitted to our cardiovascular surgery service. Previously, she had received infertility treatment with follicle-stimulating hormone, luteinizing hormone, medroxyprogesterone, clomiphene, and cetrorelix for several weeks. This was followed by a frozen embryo transfer (FET) with a total of 6 oocytes retrieved from the right and 3 from the left side on November 05, 2020. The second FET with a total of 3 blastocysts was completed on December 08, 2020. Unfortunately, 2 of the FETs had failed. According to the patient, she had received the Medigen Vaccine Biologics Corporation (MVC, Taipei City, Taipei, Taiwan) COVID-19 vaccine on August 26, 2021, and experienced right flank pain for 2 days following the vaccination and before admission. She visited Tungs’ Taichung Metro Harbor Hospital initially, and bedside renal ultrasonography showed right perirenal fluid accumulation. The patient was then admitted to the Emergency Department of Yunlin Christian Hospital for further evaluation. Physical examination revealed right costovertebral angle tenderness. Hematological testing revealed leukocytosis with a white blood cell count of 11.8 × 10^9^/L, thrombocytopenia with a platelet count of 90 × 10^3^/μL, and a D-dimer level of more than 10 × 10^3^ mg/L. No abnormalities were noted in the survey for autoimmune disease. A contrasted abdominal computed tomography (CT) scan was ordered on September 4, 2021; the results showed thrombi within the right renal veins and inferior vena cava, as well as extensive right retroperitoneal infiltration (Fig. 1). Therefore, the patient was referred to the cardiovascular surgery ward of Changhua Christian Hospital for anticoagulant treatment.

**Figure 2.**
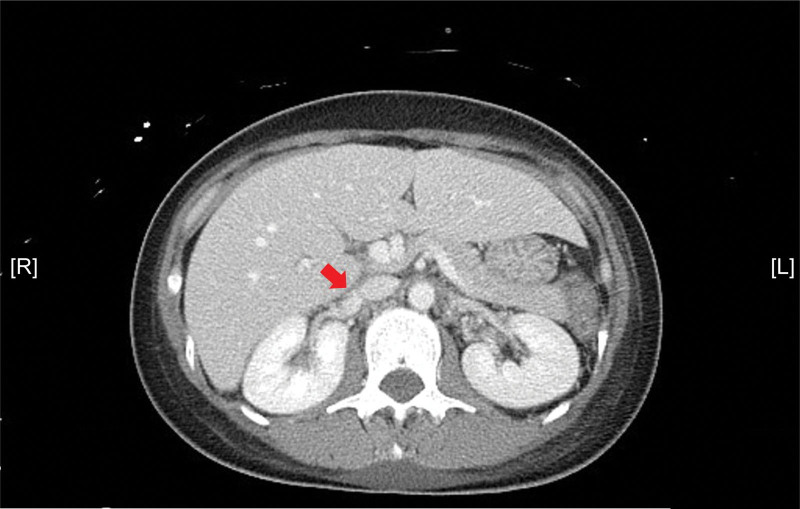
Contrasted abdominal CT images revealing improvement with approximately 90% dissolution of thrombus within the right renal vein. CT = computed tomography.

There, we performed ultrasound-assisted catheter-directed thrombolysis (EKOS Endovascular System, Boston Scientific, Natick, MA) for the patient’s right renal vein thrombus on September 07, 2021. Following the operation, an infusion of urokinase was maintained using a pump, and a fibrinogen level was checked every morning to guide the adjustment of the urokinase dosage. On the day following the procedure, the fibrinogen level was 463 mg/dL. We compounded 72 × 10^4^ units urokinase in 500 mL isotonic saline and infused at a rate of 20 mL every hour. Further, we administered 1000 mL isotonic saline to the patient for the purpose of cooling the catheter in the renal vein based on the mechanism of ultrasound-facilitated catheter-directed thrombolysis. Additionally, we prescribed 1500 mg ampicillin/sulbactam every 8 hours for 3 days to prevent septic embolism. The patient experienced dyspnea and facial edema after receiving the urokinase infusion for 2 days. Due to the suspicion of pulmonary congestion, we reduced the cooling hydration from 1000 to 500 mL daily at the suggestion of the EKOS vendor. Portable chest radiography was arranged, and pulmonary congestion was confirmed. Thus, 10 mg in-bag furosemide was administered every 12 hours, and oxygen was supplemented from 3 L/min via nasal cannula to 9 L/min via simple mask. Because the fibrinogen level was 198 mg/dL, 12 units of cryoprecipitate was transfused on September 12, 2021. An additional contrasted abdominal CT scan was scheduled on September 14, 2021, for evaluation of thrombolysis. The report revealed that 90% of the thrombus in the right renal vein was dissolved and that partial obstruction and stenosis of the left renal vein with collateral vascular formation remained (Fig. 2). Additionally, compared with findings of the previous abdominal CT, a new bilateral pleural effusion appeared, likely related to fluid infusion. Concerning the symptoms of dyspnea and edema and their slow improvement under furosemide treatment, ultrasound-guided needle aspiration was arranged on September 13, 2021. Drainage produced 500 mL transudative pleural fluid from the left side and 500 mL transudative fluid from the right side. We shifted the thrombolysis regimen to rivaroxaban 15 mg/tablet, 1 tablet per day, and discontinued the urokinase infusion 1 day later based on the improvement observed on the abdominal CT images. The final diagnosis was right RVT with renal congestion and impaired renal function. The patient was discharged in stable condition after being thoroughly educated on the potential side effects of the anticoagulant. She was assigned to the outpatient department for follow-up 7 days later and once every month. No discomfort or medication side effects were observed at the post-discharge office visit. A follow-up abdominal CT scan was arranged 3 months later, and no renal vein thrombus was observed. The treatment course of rivaroxaban lasted 5 months after consensus was reached between the doctor and the patient. Moreover, after considering the rarity of her diagnosis and clinical manifestations, the patient consented to publication of her data to raise awareness of the differential diagnosis and to promote the treatment protocol.

## 3. Discussion

This study presents the case of a young woman with primary thrombosis in the right renal vein and inferior vena cava along with a therapy protocol.

RVT is a rare condition caused by thrombus formation in the renal veins or their branches, most commonly occurring in adults with nephrotic syndrome and newborns with volume contraction or inherited thrombophilia. Symptoms include flank pain and hematuria; acute kidney injury and pulmonary embolism may eventuate based on the pathophysiology. Delayed diagnosis often occurs due to nonspecific symptoms and nonpriority of differential diagnosis. In this case, RVT was discovered incidentally based on imaging studies. The prevalence of RVT has wide variability owing to dimensions of study design, such as inclusion criteria of patients or methods of detection. Typically, most cases occur on the patient’s left side; however, in the case presented here, the RVT was on the patient’s right side with inferior vena cava involvement.^[3,8]^

Most RVT cases occur in response to a precipitating factor, such as nephrotic syndrome, trauma, oral contraceptive use, hypovolemia, or inherited thrombophilia.^[2,6,9]^ Spontaneous RVT is rare in people without nephrotic disease or an underlying renal malignancy.^[5]^ The case of spontaneous RVT reported here occurred in a patient with neither underlying pathology. We drew upon 2 aspects of her medical history that may have contributed to the condition. The first was hormone therapy for infertility. Although the interval between hormone therapy and thrombosis was approximately 8 months, previous articles have described contraceptive effects on coagulation,^[10]^ and the composition and mechanism of contraception is similar to that of infertility treatment in that both regulate follicle-stimulating hormone and luteinizing hormone levels.

The other possible etiology was the COVID-19 vaccine. As COVID-19 vaccines have been administered to an increasing number of people, their low risk for adverse events has been established.^[11]^ However, there have been reports of vaccine-induced thrombotic thrombocytopenia in the literature and 1 case of RVT following exposure to the AstraZeneca vaccine.^[12]^ Furthermore, no comprehensive systematic review has yet studied the adverse reactions to the MVC COVID-19 vaccine, with only 1 case report describing a patient with multiple evanescent white dot syndrome after receiving the vaccine.^[13]^ In the present case, the patient was administered the MVC COVID-19 vaccine 1 week before admission for RVT, and we thus reasonably infer that the thrombosis may have been associated with the vaccine.

Treatment of an acute RVT is based on the presence or absence of acute kidney injury (AKI). Usually, patients with AKI should undergo dissolution or removal of the thrombus, whereas those without AKI can be treated with therapeutic anticoagulation. The EKOS endovascular system is an interventional device for ultrasound-assisted catheter-directed thrombolysis. The use of ultrasound waves accompanied by local thrombolysis can accelerate resolution of the clot, the benefits and efficacy of which have been discussed in the previous literature. However, most cases are related to pulmonary embolism, acute aortic occlusion, or acute limb ischemia.^[14–16]^ In this case, we inserted the EKOS catheter into the patient’s right femoral vein and infused 72 × 10^4^ units urokinase in 500 mL isotonic saline at a rate of 20 mL every hour. The fibrinogen level was checked daily and the dosage adjusted according to the consensus of the attending clinicians. Blood transfusion with cryoprecipitate was given if necessary (Table 1). Moreover, we administered antibiotics to prevent thrombolytic sepsis and discontinued them after 3 days without fever or other evidence of infection. The satisfactory result in this case using the EKOS endovascular system, standard surveillance, and the urokinase protocol provides insight for the treatment of RVT. The protocol should be adjusted according to the physician’s assessment based on fluid status and renal function. Further evidence is needed regarding the efficacy and safety for patients with RVT who do not accept or are not suitable for EKOS.

**Table 1:** Protocol for the adjustment of urokinase dosage and blood transfusion with cryoprecipitate according to fibrinogen level.

Fibrinogen level (mg/dL)	*Urokinase dosage	Blood transfusion with cryoprecipitate (units)
>450 on 2 measurements	Call the attending clinician for further evaluation	Nil
450–200	Infuse at 20 mL/h	Nil
200–150	Infuse at 20 mL/h	12 units
150–100	Reduce to 10 units/h	12 units
<100	Discontinue the urokinase infusion	12 units

*72 × 10^4^ units urokinase was added to 500 mL isotonic saline.

## 4. Conclusions

This case serves as a reminder that RVT should be considered in patients with flank pain, especially after hormone therapy or vaccination. Furthermore, urokinase infusion therapy showed sufficient safety and efficacy under our proposed protocol that includes monitoring of the fibrinogen level over time.

## Acknowledgments

We would like to express great appreciation to the patient for agreeing to the publication of her experience. In addition, we extend special thanks to the staff of Changhua Christian Hospital for their excellent patient care.

## Author contributions

Treatment of patients, data collection and assessment: TSL, JYH. Manuscript writing, discussion, revision: JYH, TSL.

**Conceptualization:** Jhe-Yuan Hsu, Tzong-Shiun Li.

**Data curation:** Jhe-Yuan Hsu, Tzong-Shiun Li.

**Formal analysis:** Jhe-Yuan Hsu.

**Funding acquisition:** Tzong-Shiun Li.

**Investigation:** Jhe-Yuan Hsu.

**Methodology:** Jhe-Yuan Hsu, Tzong-Shiun Li.

**Project administration:** Jhe-Yuan Hsu.

**Resources:** Tzong-Shiun Li.

**Software:** Jhe-Yuan Hsu.

**Supervision:** Tzong-Shiun Li.

**Validation:** Jhe-Yuan Hsu.

**Visualization:** Tzong-Shiun Li.

**Writing – original draft:** Jhe-Yuan Hsu.

**Writing – review & editing:** Tzong-Shiun Li.
